# Ethnicity in neuro-oncology research: How are we doing and how can we do better?

**DOI:** 10.1007/s11060-024-04769-1

**Published:** 2024-09-24

**Authors:** Asfand Baig Mirza, Feras Fayez, Sami Rashed, Layla Burn, Zachariah M. Evans, Zekiye Karagozlu, Amisha Vastani, Jose Pedro Lavrador, Francesco Vergani, Richard Gullan, Ranjeev Bhangoo, Keyoumars Ashkan

**Affiliations:** 1 Department of Neurosurgery, Kings College Hospital, Denmark Hill, London, SE5 9RS UK; 2 Department of Neurosurgery, Queens Hospital Romford, Romford, UK; 3 Imperial College Healthcare Trust, London, UK; 4 Barts and The London School of Medicine and Dentistry, London, UK; 5 Hull Royal Infirmary, Hull, UK; 6 Department of Neurosurgery, St George’s Hospital, St George’s University Hospitals NHS Foundation Trust, London, UK

**Keywords:** Neuro-oncology, Ethnic Minorities, Clinical Trials, Systematic Review, Meta-analysis, Treatment Outcomes

## Abstract

**Purpose:**

This study systematically reviews and meta-analyses the extent of ethnic minority representation in neuro-oncology Phase III and IV clinical trials, explores the effect of ethnicity on outcomes, and identifies predictors for the inclusion of ethnicity data in publications.

**Methods:**

Adhering to PRISMA guidelines, we conducted a comprehensive literature search across multiple databases, on Phase III and IV trials in neuro-oncology that reported on adult and/or paediatric subjects. Through meta-analysis, we synthesized information on overall survival, event-free survival, and the incidence of adverse outcomes across ethnicities.

**Results:**

From 448 identified articles, a fraction reported ethnicity data, with an even smaller number providing outcome data stratified by ethnicity. Most study participants were identified as White, underscoring a significant underrepresentation of minorities. Our meta-analysis did not reveal significant outcome differences by ethnicity, which may be attributed to the limited and inadequate reporting of data. Predictors for including ethnicity data were identified, including trials in North America(OR2.39, 95%CI 1.18–5.12, *p* < 0.02),trials of drugs or biologic agents(OR 5.28, 95%CI 1.43–3.42, p < 0.05),and trials funded by charities(OR 2.28, 95% CI 1.04–5.27, *p* < 0.05) or pharmaceutical companies(OR 3.98, 95% CI 1.60–10.0, *p* < 0.005).

**Conclusion:**

The underrepresentation of minorities in neuro-oncology clinical trials and the inadequately characterized impact of ethnicity on treatment outcomes highlight a critical need for more inclusive recruitment strategies and improved reporting standards. Change is necessary to ensure trials reflect the diversity of the patient population, which is essential for developing tailored strategies and improving outcomes. Future research should prioritize understanding the role of ethnicity in neuro-oncology to facilitate personalized treatment approaches.

**Supplementary Information:**

The online version contains supplementary material available at 10.1007/s11060-024-04769-1.

## Introduction

It has long been recognised that patients in different ethnic groups respond differently to treatment [1]. Many guidelines, such as those for the management of hypertension [2], address ethnicity specifically and use evidence-based recommendations to offer patients tailored therapy for their disease and demographic [3].

There are many barriers to recruiting ethnic minorities, including language barriers, cultural constraints but also egregious abuses towards them in historical trials [4]. The Tuskegee Syphilis Study [4] is the most infamous of these but more recently both the Canadian [5] and Australian governments [6] have apologised for their part in forced experimentation on their respective indigenous peoples, testing the BCG vaccine against children in Canada and experimenting on pain tolerance in Australia.

The World Medical Association published the Declaration of Helsinki [7] as the basis for ethical human research and in 1993 the National Institutes of Health (NIH) Revitalization Act aimed to increase the number of female and ethnic minority patients in clinical trials [8]. Despite this, it is well documented that ethnic minorities have been under-represented in clinical trials and Allison et al. commented on the difficulties of doing so [4]. Disappointedly, the problem continues to persist and, for example, Duma et al. found a decrease in the recruitment of minorities to oncology clinical trials specifically from the years 2003–2016 [9].

Primary brain tumours are 1% of yearly new cancer diagnoses and lead to 2% of deaths caused by cancer [10]. Neuro-oncology trials provide a significant opportunity for patients and healthcare workers alike to increase their understanding of the disease, progress the field and improve patient outcomes and quality of life [11]. There are recognised differences in the prevalence and incidence of brain tumours in different ethnic groups [12], but the literature on outcomes and if management should differ between them is thin [13]. Both the reporting of ethnicity data in neuro-oncology trials and outcomes in different ethnic groups are critical to our understanding of treatment options and how best to tailor treatment regiments to each patient [4, 11].

There is additional urgency in the inclusion of ethnic-minority data in neuro-oncology clinical trials both because of the terrible prognosis conferred by many of the brain tumours [14] and additionally their epidemiological rarity when compared to more common conditions such as diabetes or hypertension [2]. Trials with more participants have stronger statistical power [15] and therefore it is important to recruit ethnic minority patients both so that they may be better represented [4] and to increase the overall power of the trials.

Taha et al. analysed brain tumour trials registered on www.clinicaltrials.gov between the years 2005 and 2017 and found that despite their increasing numbers in the United States, ethnic minorities remained under-represented and notwithstanding the legislation and recommendations, many trials did not publish mandatory data on race or ethnicity [11]. Here we present data with a global perspective to systematically review and meta-analyse all available Phase III and IV neuro-oncology trials with the primary aim to assess the extent to which ethnic minorities are represented. A secondary aim is to explore the extent to which ethnicity affects responsiveness to therapy in terms of overall survival (OS), event free survival (EFS) and adverse outcomes/side effects. Finally, an analysis of factors that predict inclusion of ethnicity data will be performed including year, trial location and source of funding.

## Methods

We conducted a meta-analysis and systematic review of randomized control trials (RCTs) on subjects with primary or secondary intracranial tumours. This was performed as per the Preferred Reporting Items for Systematic Reviews and Meta-Analyses (PRISMA) guidelines [16].

### Search strategy

PubMed, OVID Medline, EMBASE, and Cochrane Library databases were used for a comprehensive and systematic literature search, using related terms “Neuro-Oncology”, “Brain tumour”, “RCT”, and variations thereof. Search and screening were conducted up to January 25, 2024.

### Inclusion criteria

Eligible articles included any phase III and IV RCT in Neuro-Oncology, published in English language with adult (18 +) and/or paediatric human subjects, with centres in any continent. RCTs were included with any primary or secondary CNS tumour type and any intervention type. All outcomes were included.

Full text articles which met the inclusion criteria (known as All RCTs) were screened for whether patient ethnicity data was included. Those articles which describe the different ethnic groups included within their trial cohort are referred to as Demographic RCTs herein and those articles which included data on how trial outcomes were affected by ethnicity are referred to as Outcome RCTs.

### Exclusion criteria

Phase I or II RCT and any non-randomised control trial were excluded. Trials of spinal cord tumours or other non-neurological tumours were excluded in an attempt to reduce heterogeneity and increase reliability and reproducibility of the data.

Full text articles which met the inclusion criteria (known as All RCTs) were screened for whether patient ethnicity data was included. Those articles which describe the different ethnic groups included within their trial cohort are referred to as Demographic RCTs herein and those articles which included data on how trial outcomes were affected by ethnicity are referred to as Outcome RCTs.

### Risk of bias

We used the NHLBI Quality Assessment Tool for Controlled Intervention Studies [17] to assess for low validity of results or any subsequent bias. If there was a high risk of bias, they were excluded after an independent review by senior authors.

### Primary analysis

Baseline data pertaining to trial start date, geographical location, tumour type investigated, intervention investigated, and sources of funding were extracted for all articles by three independent authors. These baseline characteristics between groups were assessed for independence using Fisher’s exact test as > 20% of data had expected values < 5. Those with a p-value < 0.05 were considered significant. Significant differences between the groups were further analysed through multiple computational pairwise Fisher’s exact test with Bonferroni correction to identify the underlying cause of significant differences between groups.

Outcome RCTs then had raw data related to primary study outcomes broken down by ethnicity extracted where available. Analogous endpoints to those defined in the original articles were used for meta-analysis; OS, defined as time of randomisation to death, and EFS defined as disease progression, recurrence, or death from any cause. Meta-analysis of outcome RCT data was then performed using the Mantel-Haneszel method for those articles which compared intervention and control groups. For studies which only described ethnicity outcome data as a composite of both intervention and control groups, proportional meta-analysis was employed using random intercept logistic regression model and logit transformation. Both fixed and random effects modelling was used depending on study heterogeneity identified by Q, I^2^ and restricted maximum-likelihood estimator for tau.

Ethnic Groups included White i.e. from Caucasian descent, Black i.e. from African or Caribbean descent, Hispanic i.e. from Latin American descent, Asian i.e. from East and South Asian descent and Other, which included a composite of less well represented ethnicities in the literature such as Arab, Middle Eastern, Hawaiian descent. Ethnicity data broken down by individual ethnic groups was used in preference for summative analysis however this data was converted to the larger subgroups of white vs non-white ethnic groups to allow summation of data with articles which only used this ethnicity distinction in their original text.

### Secondary analysis

Multivariate logistic regression analysis was employed to investigate baseline characteristics positively associated with the inclusion of ethnicity demographic and outcome data. Odds ratios, 95% Confidence Intervals (CI) and p-values were also calculated.

All statistical analysis was performed in RStudio Version 2023.09.1 + 494.

## Results

### Baseline demographics

**Fig. 1:**
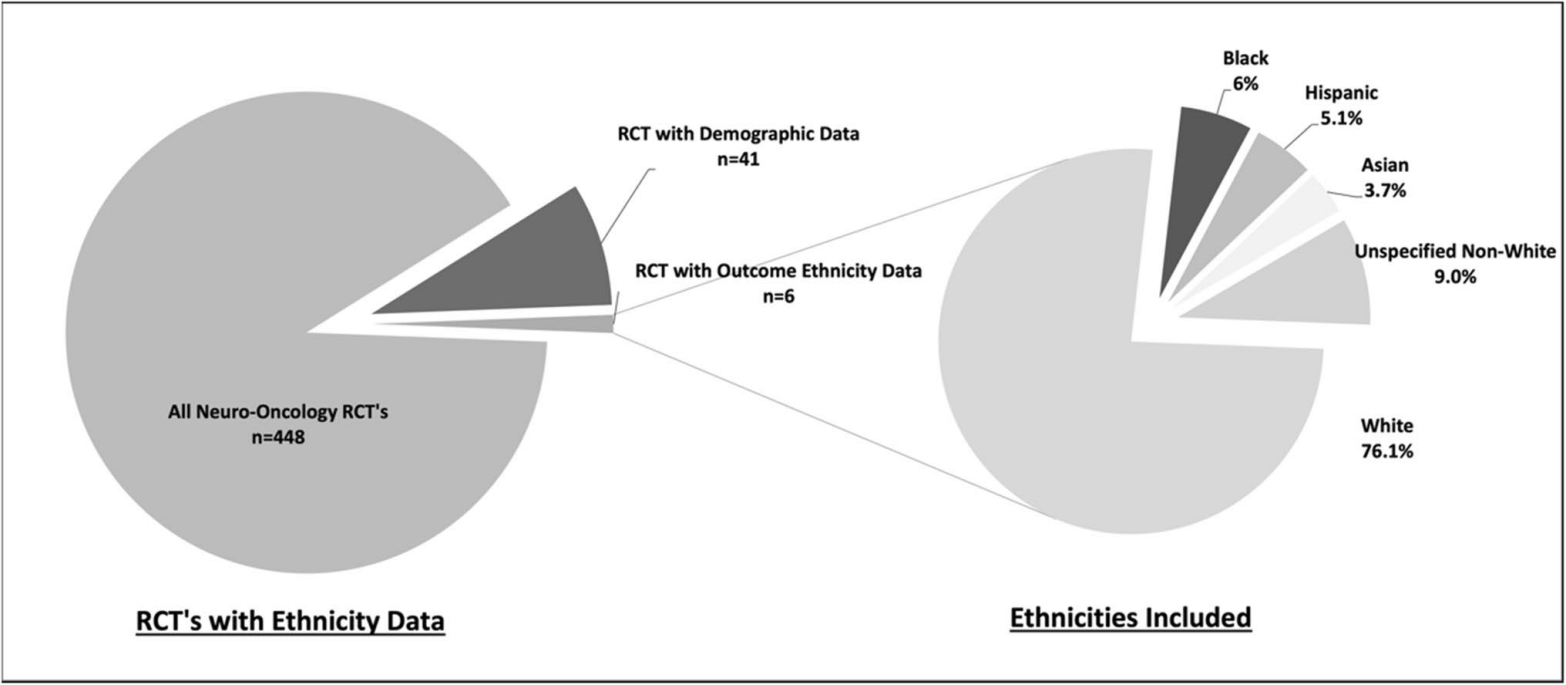
All neuro-oncology trials broken down by the inclusion of ethnicity data (left) and the proportions of ethnicities included in these trials (right)

A total of 448 articles met inclusion criteria for a RCT conducted in the field of neuro-oncology with 42 describing demographic ethnicity data and 6 describing outcome data in terms of ethnicity (Supplementary Table 1). Of the articles describing outcome ethnicity data included in quantitative analysis, a total of 2024 participants were described by race. Of these 1541 (76.1%) were described as White, 121 (6.0%) were Black, 104 (5.1%) were Hispanic, 75 (3.7%) were Asian and 183 (9.0%) were described as an Unspecified Non-White in the original text (Fig. 1).

Baseline characteristics of All RCTs, Demographic RCTs and Outcome RCTs were assessed; a significant difference in trial geographical origin (*p* < 0.001) and the intervention type studied (*p* < 0.002) was found on initial testing. Further analysis found RCTs conducted in North America to be significantly more likely to include demographic data (*p* < 0.05) and studies designed to investigate a novel drug/biologic (*p* < 0.002) compared with All RCTs were also more likely to report demographic ethnicity data. (Supplemental Figs. 2B&C). No significant difference found for year of trial start date (*p* = 0.375), tumour type examined (*p* = 0.461) and funding source between groups (*p* = 0.107) (Supplemental Fig. 2A).

### Primary analysis

A total of 6 articles identified described the effect of ethnicity on any type of outcome data. Two articles only described the effect of ethnicity through the output of their regression analysis and so raw data was not available for summative assessment. A total of 4 articles were suitable for quantitative meta-analysis [18–21] and their baseline characteristics can be seen in Table 1. Two articles described the effect of novel biologic/drugs in the adult GBM population whereas the other two articles described the effect of chemo/radiotherapy regimes in a paediatric medulloblastoma cohort. Median follow up was 55.5 months (4.6 years).

**Table 1:** Study characteristics of those articles included in quantitative Meta—analysis of neuro-oncology outcomes by ethnicity

Study	Year	Continent	n	Population	Age	Male	Ethnicity (n) Included in total	Inclusion Criteria	Intervention	Primary Outcomes	Follow Up
Lim et al. 2022 [18]	2016	North America	716	Adult	Median 60	402 56%	White 619 Black 8 Asian 68 Other 20	GBM newly diagnosed grade IV on histology undergoing surgical resection	Following standard care randomised to checkpoint inhibitor nivolumab (NIVO) vs placebo	- Primary endpoint progression free survival - Overall survival	4.5 years
Tarbell et al. 2013 [19]	1990	North America	224	Paediatric	Range 3–21 Median 7.8	132 59%	White 171 Black 34 Hispanic 12 Asian 7	High risk Medulloblastoma risk undergoing surgical resection	Randomisation to receive chemotherapy before radiation of chemotherapy after radiation	- Event free survival (EFS) - Overall Survival	5- Years
Weller et al. 2017 [20]	2012	North America	743	Adult	Median 58.5	480 64%	White 668 Other 75	Newly diagnosed GBM expressing EGFR undergoing surgical resection	Standard care randomised to either Rindopepimut (EGFR targeting vaccine) or placebo	- Overall Survival	4 years
Packer et al. 2006 [21]	1996	North America	342	Paediatric	Range 3–19	223 59%	White 83 Black 79 Hispanic 92 Other 88	Histologically confirmed Medulloblastoma with no dissemination of disease undergoing surgical resection	Randomisation to one of two experimental regimens of radiotherapy plus one or two adjuvant chemotherapy regimens	- Treatment failure event	5 years

*N* number, *GM* Glioblastoma, *EGFR*  Epidermal Growth Factor Receptor

Of the four included articles for quantitative analysis a total of 2024 participants were described by race. Of these 1541 (76.1%) were described as white with 483 (23.9%) were described as Non-White (Fig. 1). Three articles described individual ethnicities whereas one article only described white vs non-white ethnicity cohorts.

### Overall survival

**Fig. 2:**
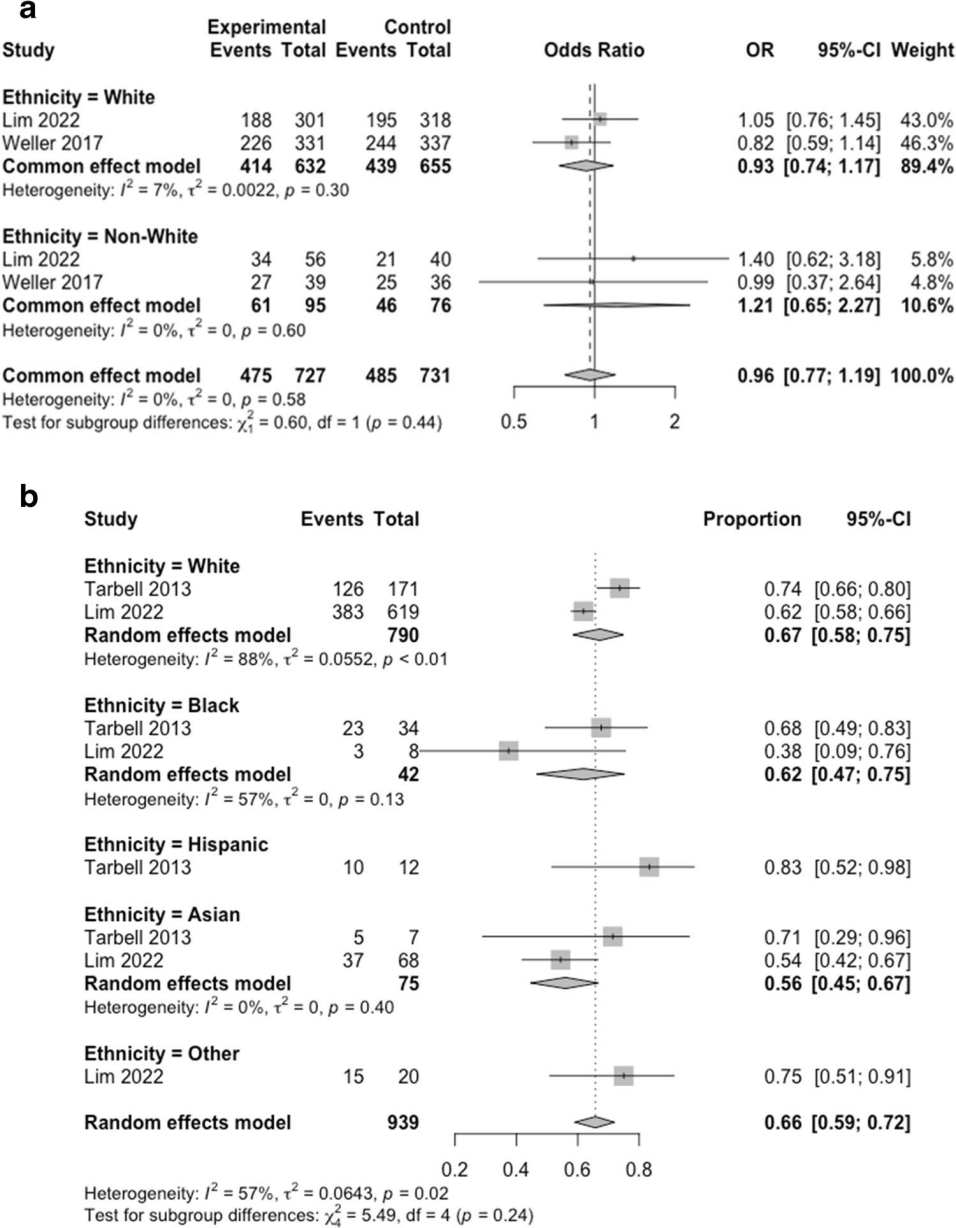
Effect of Ethnicity on Overall Survival; Comparing Intervention Against Control (**A**) and Comparing Intervention and Control Groups Irrespective of Randomisation (**B**)

Two articles, reporting the effect of a biologic/drug in adult GBM, described the effect of the intervention against the control group on OS in different ethnicities with no significant heterogeneity between studies (I^2^ = 0%, τ^2^ = 0, *p* = 0.58). Pooled ORs of whites vs non-whites showed a trend for favourable survival rates in non-whites who received the intervention (OR 0.93 (95% CI 0.74 – 1.17) vs OR 1.21 (95% CI 0.65–2.27) however this was not significant (X^2^, df = 1, p0.44) (Fig. 2A).

Two articles described overall survival in ethnic groups as a composite of both intervention and control groups. Significant heterogeneity was present between these (I^2^ = 57%, τ^2^ = 0.064, *p* = 0.02). Pooled odds of OS were 0.67 in white (95% CI 0.58–075), 0.62 in Black (95% CI 0.47–0.75), 0.83 in Hispanic (95% CI 0.52–0.98), 0.56 in Asian (95% CI 0.45–0.67) and 0.75 (95% CI 0.51–0.91) in Other un-specified ethnicity cohorts. These differences were however non-significant (X^2^, = 5.49 df = 4, p0.24) (Fig. 2B).

### Event free survival

Three articles described EFS across ethnic groups without significant heterogeneity between them (I^2^ = 39%, τ^2^ = 0.0368, *p* = 0.08).

**Fig. 3:**
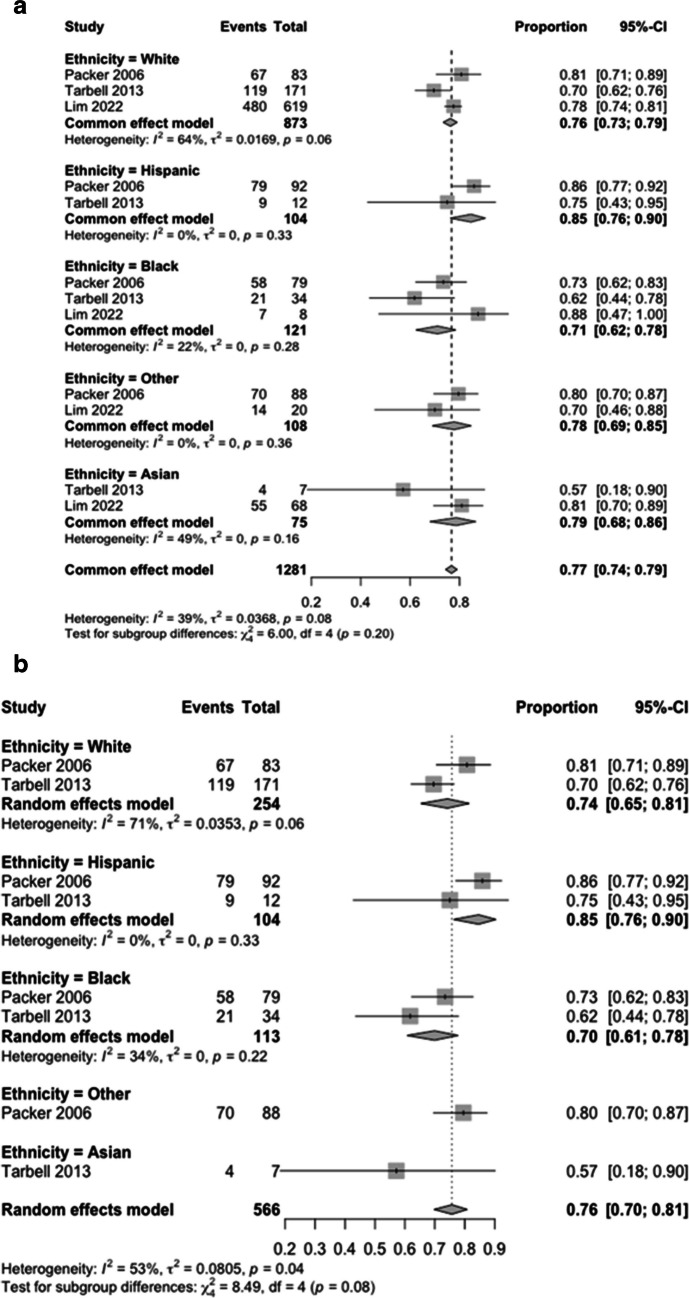
Effect of Ethnicity on Event-Free Survival; In Intervention and Control Groups Irrespective of Randomisation (**A**) and in the Paediatric Specific Medulloblastoma Cohort irrespective of Randomisation (**B**)

Pooled odds of EFS were 0.76 in White (95% CI 0.73–079), 0.71 in Black (95% CI 0.62–0.78), 0.85 in Hispanic (95% CI 0.76–0.90), 0.79 in Asian (95% CI 0.68–0.86) and 0.78 (95% CI 0.69–0.85) for Other un-specified ethnicity cohorts. These differences were however non-significant (X^2^, = 6.00, df = 4, p0.2) (Fig. 3A).

Two articles described the effect of radiation treatment in a paediatric cohort. There was significant heterogeneity between the groups however (I^2^ = 53%, τ^2^ = 0.081, *p* = 0.04). Pooled odds of EFS were 0.74 (95% CI 0.65–0.81) in white, 0.85 in Hispanic (95% CI 0.76 – 0.9), 0.70 in Black (95% CI 0.61 – 0.78), 0.57 in Asian (95% CI 0.16 – 0.9) and 0.8 (95% CI 0.7 – 0.87) in the non-specified ethnicity cohort. Of note, these differences were approaching significance (X^2^, = 8.49, df = 4, p0.08) (Fig. 3B).

All four studies documented toxicity and side effect data however no article broke down this data to assess if unequal distributions existed between ethnic groups.

### Secondary analysis

Multivariate logistic regression was then performed to assess for article features which were positively or negatively predictive of including ethnicity demographic or outcome data (Table 1.). Significant factors found to be positively predictive of the inclusion of ethnicity demographic data were, publication decade between 1984–94 (OR 3.08, 95% CI 0.04–4.33, *p* < 0.05), 2004–14 (OR 2.57, 95% CI 1.09–6.24, *p* < 0.05), Trial continent of North America (OR2.39, 95% CI 1.18–5.12, *p* < 0.02), intervention investigated: Drug or Biologic (OR 5.28, 1.43–3.42, *p* < 0.05) and funding received from Pharmaceutical company (OR 3.98, 95% CI 1.60–10.0, *p* < 0.005) or Cancer or Charitable Institute (OR 2.28, 95% CI 1.04–5.27, *p* < 0.05). The remaining factors and all those related to ethnicity outcome data did not show any significant negative or positive correlation (Table 2 & Supplementary Table 2).

**Table 2:** Multivariate logistic regression output for factor predictive of demographic ethnicity data inclusion

Factor Predictive of Ethnicity Demographic Data	Coefficient	SE	*Z*-Value	OR	95% CI	*P*-Value
Year						
74 – 84	-0.2563	1.0718	-0.239	0.77	0.041—4.33	0.8110
84—94	**1.1252**	**0.4858**	**2.316**	**3.08**	**1.17—8.04**	0.0205*
94—04	0.4663	0.5359	0.870	1.59	0.52—4.47	0.3843
04—14	**0.9443**	**0.4401**	**2.146**	**2.57**	**1.09—6.24**	0.0319*
14—24	-0.2343	0.7922	-0.296	0.79	0.12—3.14	0.7674
Continent						
Africa	-16.0140	3765.8	-0.004	1.11e-07	NA-1.08e + 206	0.9966
Asia	-16.0140	1044.5	-0.015	1.11e-07	2.61e-149 -7.15e + 13	0.9878
Australasia	2.5520	1.4456	1.765	12.83	0.49—338.54	0.0775
Europe	-1.1200	0.6570	-1.705	0.33	731.31—1.05	0.0882
North America	**0.8731**	**0.3705**	**2.356**	**2.39**	**1.18—5.12**	0.0185 *
South America	-16.014	4612.2	-0.003	1.11e-07	NA -1.42e + 307	0.9972
Middle East	-16.014	3261.3	-0.005	1.11e-07	NA- 1.04e + 155	0.9961
Tumour Type						
Glioblastoma	-0.2933	0.5302	-0.553	0.75	0.27—2.26	0.580
Astrocytoma	-0.6817	0.8508	- 0. 0.801	0.51	0.071- 2.37	0.423
Lymphoma	-15.573	2284.1	- 0.007	1.72e-07	NA—1.01e + 122	0.995
Oligodendroglioma	-15.573	959.51	- 0.016	1.72e-07	2.60e-137 -2.55e + 12	0.987
High Grade Glioma	-0.03195	0.59255	- 0.054	0.97	0.30—3.21	0.957
Low Grade Glioma	-0.61026	0.85231	- 0.716	0.54	0.076—2.55	0.474
Ependymoma	- 15.574	2797.4	- 0.006	1.72e-07	NA—2.61e + 183	0.996
Medulloblastoma	- 0.8979	1.1158	- 0.805	0.41	0.021—2.62	0.421
Metastasis	- 0.5531	0.56938	- 0.971	0.57	0.19—1.84	0.331
Pituitary	- 15.573	3956.18	- 0.004	1.72e-07	NA—Infinity	0.997
Any Supratentorial	- 15.573	1769.26	-0.009	1.72e-07	NA—8.32e + 71	0.993
Any Primary CNS	- 0.4924	0.74196	- 0.664	0.61	0.12—2.49	0.507
Intervention						
Behavioural	- 15.5703	3765.85	-0.004	1.73e-07	0.00—1.93e + 68	0.9967
Genetic	- 15.5703	1630.66	-0.010	1.73e-07	1.45e-228—5.42e + 25	0.9924
Procedure	- 15.5703	1630.66	-0.010	1.73e-07	6.40e-216—7.51e + 23	0.9919
Drug or Biologic	**1.6635**	**0.7691**	**2.163**	**5.28**	**1.43—34.20**	0.0305*
Radiation	0.1300	0.7688	1.003	1.14	0.31—7.37	0.3161
Surgery & Radiation	1.2910	1.0564	0.169	3.64	0.40—33.27	0.8658
Other	1.0498	1.0471	1.222	2.86	0.32—25.72	0.2217
Funding						
Cancer or Charitable Foundation	**0.8248**	**0.4086**	**2.019**	**2.28**	**1.04—5.27**	0.04351*
Pharmaceutical Company	**1.3813**	**0.4627**	**2.985**	**3.98**	**1.60 – 10.03**	0.00283**
University	-13.5753	723.4900	-0.019	1.27e-06	1.47e-104—3.54e + 08	0.98503
Other	0.1874	0.7971	0.235	1.206061e + 00	0.18—4.84	0.81416

*CNS* Central Nervous System, *NA* Not Applicable

## Discussion

RCTs are a critical tool in our fight against cancer. In the field of neuro-oncology they are even more precious due to the epidemiological rarity associated with the diseases and the terrible prognoses conferred by them. We believe this literature review and meta-analysis is critical to the literature as it highlights the reporting standards that demographic data, outcomes, and negative effects are published as. It additionally identifies factors predictive of including ethnicity data in publication. Overall, our study found very few RCTs that reported demographic ethnicity data, fewer still that reported outcomes by ethnicity and none that reported negative effects of intervention by ethnicity.

### Ethnicity data

As part of our inclusion criteria studies needed to report outcomes to be included. Of the 448 studies, all of which reported outcomes, only 42(9.4%) even mentioned demographic data regarding ethnicity. Taha et al. [11] found that 27% of trials in their search reported no outcomes and of the 73% that did, only 28% reported any race information at all. We have focused on randomised phase III and IV trials with outcomes, and we find an even fewer number of trials reporting the demographics of their studies. Furthermore, only 6 (1.3%) trials reported outcomes by ethnicity. Given that many trials do not release their raw data, there is therefore much data that could contribute to the literature that is not being utilised. Only 13% of the studies with demographic data provided outcomes linked to these demographics, a simple step given these participants already have outcomes recorded, which would contribute to our understanding of neuro-oncology in minorities.

### Lack of diversity

Of the few trials that have reported ethnicity data, there is very little specificity in the reporting of ethnicities. “White” is described as the most common ethnicity at 76% but this has been identified as a misnomer [22]. The use of the term white is typically used to refer to participants of European origin of a pale complexion, yet white can also encompass ethnicities such as Turkish, Middle Eastern [23], North African and even parts of west Asia under the definition used by the United States Census Burea [24, 25]. This is a diverse group of people to label as “white” and it is possible cross-sectional studies may reveal differences in outcomes and adverse effects if done [26]. Additionally, broad labels like “Asian” [27], can encompass multiple ethnic groups. In one study analysing the reporting of demographics they identified 263 articles that stratified patients into the “Asian” demographic [27]. Of these studies only 9 (3.4%) differentiated this into different Asian demographics [27]. Overall, very few studies are reporting any demographic data and even in those that do, this is done to a poor standard.

### Socioeconomics

Low income and general socioeconomic class has previously been identified as a potential factor leading to worse neuro-oncological outcomes in the literature [28–30]. Ethnic minorities are often over-represented among low income communities [31] and future research must ascertain whether it is ethnicity or socioeconomic status that leads to these reported outcomes.

### Meta-analysis – differences in ethnic outcomes

Of 6 studies that did include outcome data, only 4 provided raw data and were included in our meta-analysis of the effect of ethnicity on OS as well as the effect on EFS. No significance was found between the small number of studies in the any populations, GBM or medulloblastoma, biologic treatment or radiation. Studies have found significant differences in incidence [30], treatment decisions [32], post-operative complications [30, 32, 33] and outcomes [1, 12–14, 24, 30, 32–35] in patients of different ethnicities and therefore, it is likely that the small number of Phase III and IV trials that reported ethnicity outcomes do not have the statistical power required to reach significance levels in this meta-analysis though there were some trends (positive and negative) with one approaching significance. The lack of data combined with the large skew of distribution in the few that did include ethnicity data towards white patients (76%) limits the power of this analysis to truly find any differences or rule them out. This needs to be addressed in larger studies that both report ethnicity outcomes and ensure a large enough representation of ethnic minorities to truly identify statistically significant trends.

### Negative effects of intervention

A key finding in our review, is that no trials reported negative effects of intervention by ethnicity despite all trials reporting negative outcomes. Studies have found that patients from ethnic minorities were likely to have extended length of hospital stay compared to Caucasian patients [32, 36], additionally more incidence of cardiovascular complications [36] and complications in general are seen in the former groups [32]. In north American studies it has been reported that African-Americans were more likely to have non-home discharge disposition [37, 38] and more common 90-day post-surgical re-admission [39]. Given these studies, it is critical that trials report negative effects by ethnicity or race. Several studies have identified adverse outcomes and side effects as a barrier to ethnic minority participation in RCTs [4, 40–42]. This may in part be due to the lack of evidence that interventions are/are not safe for these populations. When 76% of trial participants are labelled as “white”, ethnic minorities may not feel the studies are representative of how they may respond to the trialled therapies. Historically, one has seen the importance of trialling medications in a diverse range of demographic populations. Tragedies like thalidomide [43] have highlighted the dangers of not thoroughly testing a drug and while standards for approving drugs have improved, it is important we stay vigilant.

### Predictors of inclusion of ethnicity data

Trials conducted in North America were most likely to include ethnicity data. Of the trials reporting ethnicity demographic data in this review, 90% occurred in North America and all with outcome data occurred in North America. This may in part be due to legal reporting requirements and recommendations by societies such as the NIH [44] where funding is conditional on reporting standards for women and ethnic minorities being met [8, 44, 45]. Despite this, many North American trials are still not reporting this data. Similar legislation in other jurisdictions and continents may promote more trials to include this data. Other factors identified include trials regarding novel drug or biologic therapy and funding by pharmaceutical companies or cancer/charitable foundations. Of note, 81% of trials that declared funding source were funded by pharmaceutical companies or cancer/charitable foundations and it is therefore difficult to derive true trends with such an imbalance. Surprisingly, however, no studies funded by universities included ethnicity data as a demographic or in outcome reporting.

## Conclusion

Neuro-oncology clinical trials are critically important in furthering our understanding of and ability to fight brain tumours. Their epidemiological rarity, severity and impact on patients and carers alike make these trials even more precious and important to conduct to a very high standard. In the age of personalised medicine and cancer care, it is rather surprising that very few trials are publishing data on participant ethnicity and those that are, report it to a low standard. The available data for phase 3 and 4 trials indicate that the ethnic minority participation rates remain extremely low. Recruitment to all trails is always a challenge and low participation of those from the minority ethnic backgrounds is undoubtedly multifactorial in nature. Nonetheless more work is needed to encourage ethnic minorities to participate in these trials and to report the data in a structured fashion in the future. In our view this is a mandatory step towards optimal stratified medicine for all trials from smaller phase 1 and 2 trials, to larger randomised phase 3 and 4 trials.

## Supplementary Information

Below is the link to the electronic supplementary material.

Supplementary file1 Supplementary Table 1 Baseline characteristics of articles included in this review. RCT = Randomised Control Trial, CNS = Central Nervous System, HGG = High Grade Glioma, LGG = Low Grade Glioma. Supplementary Table 2 Output from Logistic Regression of Outcome Regression (DOCX 18 KB)

Supplementary file2 Supplementary Figure 1 Prisma diagram. Supplementary Figure 2. Baseline characteristics of articles broken down by ethnicity data type incluidng trail start year, tumour types included and funding for studies (A), continent (B) and intervention type studies (C). RCT= Randomised Control Trial, GBM=Glioblastoma, HGG = High Grade Glioma, LGG = LowGrade Glioma, Pharma = Pharmacuetical Company, PF=Fisher’s Exact Test, Pb=Pairwise Fishers Test with Bonferroni Correction (DOCX 1092 KB)

## Data Availability

No datasets were generated or analysed during the current study.
