# Supplementary material for: Ethnicity in neuro-oncology research: How are we doing and how can we do better?
Source: J Neurooncol. 2024 Sep 24;170(2):223–33. doi: 10.1007/s11060-024-04769-1 (PMC11538236; doi:10.1007/s11060-024-04769-1)
Supplement: Supplementary file 1 — Supplementary file1 Supplementary Table 1 Baseline characteristics of articles included in this review. RCT = Randomised Control Trial, CNS = Central Nervous System, HGG = High Grade Glioma, LGG = Low Grade Glioma. Supplementary Table 2 Output from Logistic Regression of Outcome Regression (DOCX 18 KB) [file 11060_2024_4769_MOESM1_ESM.docx]

Supplementary table 1.

| **Article type** | **n** | **Trial Start Date (n=270)** | **Continent (n=297)** | **Tumour Type**  **(n=404)** | **Intervention**  **(n=408)** | **Funding**  **(n=251)** |
| --- | --- | --- | --- | --- | --- | --- |
| All RCT | 448 | Range 1976 – 2022  Mean 2001 | Asia n = 39  Africa n=3  South America n = 2  North America n=130  Europe n=118  Australasia n=1  Middle East n=4 | Any primary CNS tumour = 36  Astrocytoma = 27  CNS Lymphoma = 3  Ependymoma = 2  GBM = 116  HGG (WHO III/IV) = 53  LGG (WHOI/II) = 26  Medulloblastoma =16  Metastasis = 102  Oligodendroglioma = 17  Pituitary = 1  Any supratentorial tumour = 5 | Behavioural = 3  Genetic = 16  Novel Drug/Biologic Agent = 69  Other = 14  Procedure (surgery/intervention) = 17  Radiation (chemo/radiotherapy) = 278  Surgery + radiation = 11 | Cancer Institute/Charity = 156  Other = 31  Pharmaceutical company = 53  University = 11 |
| **Article Type** | **n** | **Trial Start Date (n=31)** | **Continent (n=29)** | **Tumour Type (n=35)** | **Intervention (n=39)** | **Funding (n=31)** |
| Demographic RCT | 41 | Range 1983 – 2017  Mean 2000 | North America n= 25  Europe n= 3  Australasia n= 1 | Any primary CNS tumour = 3  Astrocytoma = 2  GBM = 12  HGG (WHO III/IV) = 7  LGG (WHOI/II) = 2  Medulloblastoma = 1  Metastasis = 8 | Novel Drug/Biologic Agent = 19  Other = 2  Radiation (chemo/radiotherapy) = 16  Surgery + radiation = 2 | Cancer Institute/Charity = 18  Other = 2  Pharmaceutical company = 11 |
| **Article Type** | **n** | **Trial Start Date (n=6)** | **Continent (n=4)** | **Tumour Type (n=6)** | **Intervention (n=6)** | **Funding (n=5)** |
| Outcome RCT | 6 | Range 1976 – 2020  Mean 1999 | North America n= 4 | Astrocytoma = 2  GBM = 2  Medulloblastoma = 2 | Novel Drug/Biologic Agent = 2  Procedure (surgery/intervention) = 1  Radiation (chemo/radiotherapy) = 3 | Cancer Institute/Charity = 1  Other = 2  Pharmaceutical company = 2 |

Supplementary Table 2.

|  |  |
| --- | --- |
| **Year** Estimate Std. Error z value Pr(>\|z\|) | |
| Year74to841 18.43 2131.99 0.009 0.993 | |
| Year84to941 18.18 2131.99 0.009 0.993 | |
| Year94to041 17.29 2131.99 0.008 0.994 | |
| Year04to141 16.94 2131.99 0.008 0.994 | |
| Year14to241 18.45 2131.99 0.009 0.993 | |
|  | |
| **Continent** | |
| Africa1 -17.5709 16877.3560 -0.001 0.999 | |
| Asia1 -17.5709 4680.9364 -0.004 0.997 | |
| Australasia1 -17.5709 20670.4551 -0.001 0.999 | |
| Europe1 -17.5709 2657.4944 -0.007 0.995 | |
| NorthAmerica1 0.3380 0.7720 0.438 0.661 | |
| SouthAmerica1 -17.5709 20670.4551 -0.001 0.999 | |
| MiddleEast1 -17.5709 14616.2190 -0.001 0.999 | |
|  | |
| **Tumour Type** | |
| GBM1 -0.29335 0.53028 -0.553 0.580 | |
| Astro1 -0.68172 0.85081 -0.801 0.423 | |
| Lymphoma1 -15.57364 2284.10182 -0.007 0.995 | |
| Oligo1 -15.57364 959.51477 -0.016 0.987 | |
| HGG1 -0.03195 0.59255 -0.054 0.957 | |
| LGG1 -0.61026 0.85231 -0.716 0.474 | |
| Ependymoma1 -15.57364 2797.44197 -0.006 0.996 | |
| Medulloblastoma1 -0.89794 1.11577 -0.805 0.421 | |
| Met1 -0.55310 0.56938 -0.971 0.331 | |
| Pituitary1 -15.57364 3956.18035 -0.004 0.997 | |
| SupratentorialAny1 -15.57364 1769.25768 -0.009 0.993 | |
| TumourAnyPrimaryCNS1 -0.49248 0.74196 -0.664 0.507 | |
|  | |
| **Intervention Type** | |
| InterventionBehavioural1 4.401e-09 1.060e+04 0.000 1.000 | |
| Genetic1 5.206e-09 5.209e+03 0.000 1.000 | |
| Procedure1 1.773e+01 2.736e+03 0.006 0.995 | |
| DrugOrBiologic1 1.719e+01 2.736e+03 0.006 0.995 | |
| Other...311 5.253e-09 5.209e+03 0.000 1.000 | |
| Radiation1 1.598e+01 2.736e+03 0.006 0.995 | |
| SurgeryAndRadiation1 5.266e-09 5.627e+03 0.000 1.000 | |
|  | |
| **Funding** | |
| FundingCancerOrCharity1 -0.5195 1.2291 -0.423 0.6725 | |
| Other...361 1.8362 1.0174 1.805 0.0711 . | |
| Pharma1 1.1738 1.0102 1.162 0.2452 | |
| University1 -13.9265 1966.6497 -0.007 0.9943 | |
