# Supplementary figures and images for: Ethnicity in neuro-oncology research: How are we doing and how can we do better?
Source: J Neurooncol. 2024 Sep 24;170(2):223–33. doi: 10.1007/s11060-024-04769-1 (PMC11538236; doi:10.1007/s11060-024-04769-1)

Supplemental Figure 1


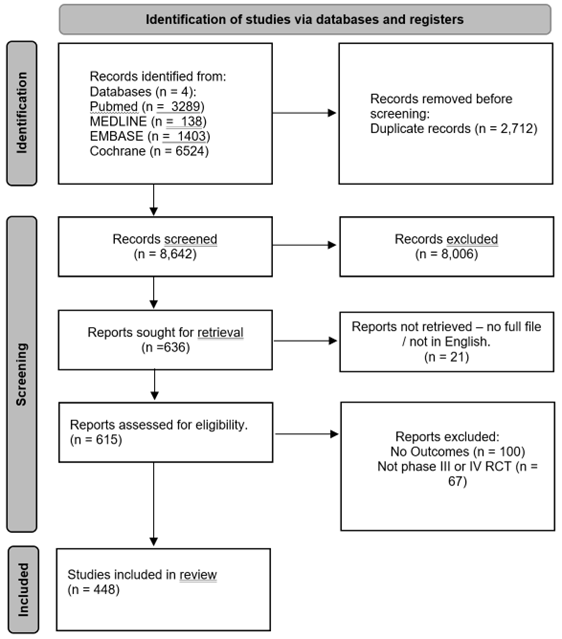


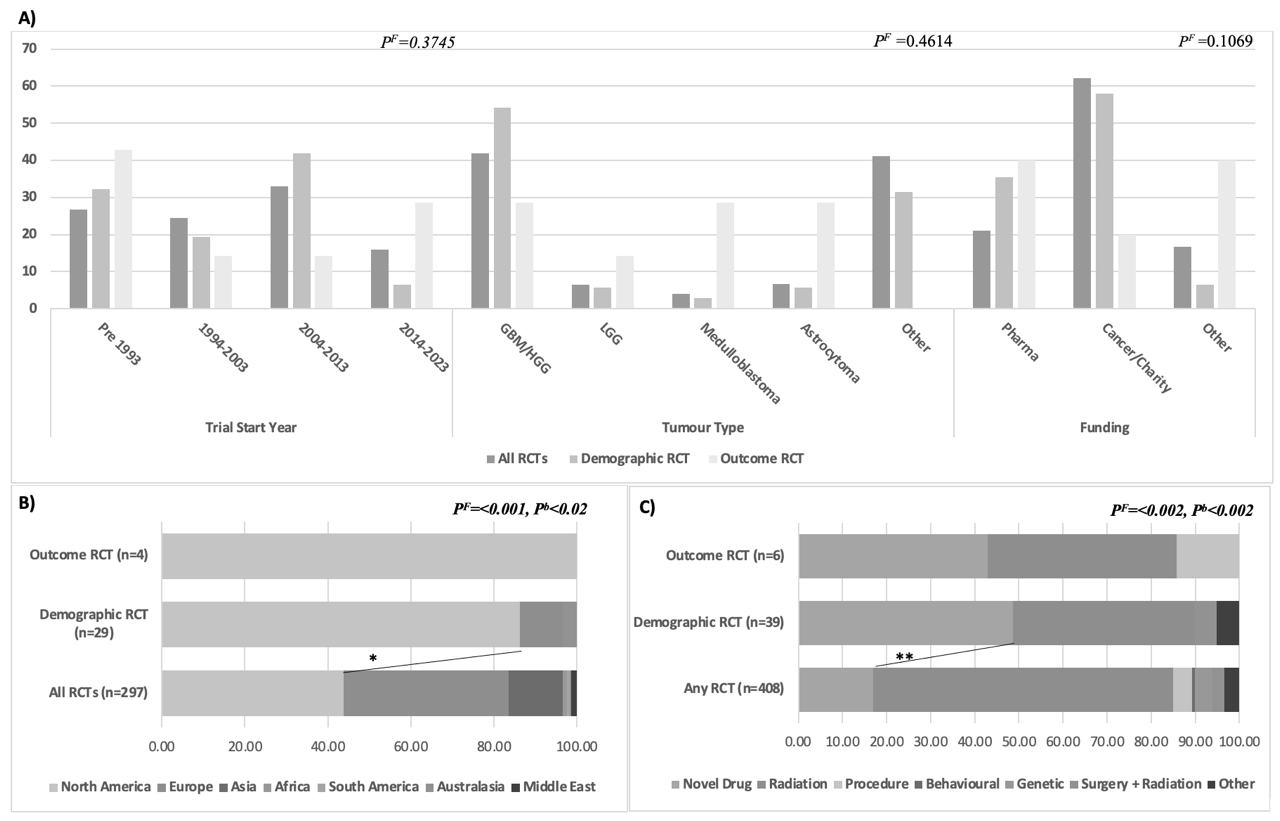
Supplemental Figure 2

Supplement: Supplementary file 2 — Supplementary file2 Supplementary Figure 1 Prisma diagram. Supplementary Figure 2. Baseline characteristics of articles broken down by ethnicity data type incluidng trail start year, tumour types included and funding for studies (A), continent (B) and intervention type studies (C). RCT= Randomised Control Trial, GBM=Glioblastoma, HGG = High Grade Glioma, LGG = LowGrade Glioma, Pharma = Pharmacuetical Company, PF=Fisher’s Exact Test, Pb=Pairwise Fishers Test with Bonferroni Correction (DOCX 1092 KB) [file 11060_2024_4769_MOESM2_ESM.docx]
